# Inclusion of Speckle Tracking Echocardiography Analysis in the Management of Intrauterine Growth Restrictions—Literature Review and Case Reports

**DOI:** 10.3390/jcm14093099

**Published:** 2025-04-30

**Authors:** Adrian Valeriu Neacșu, Adina-Elena Nenciu, Șerban Nastasia, Oana-Eliza Crețu, Alina-Alexandra Dîrlău, Iuliana Ceaușu

**Affiliations:** 1 Department of Obstetrics and Gynecology, Faculty of Medicine, “Carol Davila” University of Medicine and Pharmacy, 020021 Bucharest, Romania; adrian-valeriu.neacsu@drd.umfcd.ro (A.V.N.); adina-elena.afloarea@drd.umfcd.ro (A.-E.N.); iuliana.ceausu@umfcd.ro (I.C.); 2 Department of Obstetrics and Gynaecology, “Carol Davila” University of Medicine and Pharmacy, “Dr I. Cantacuzino” Hospital, 020021 Bucharest, Romania

**Keywords:** intrauterine growth restriction ultrasound markers, speckle tracking fetal echocardiography, pregnancy outcomes

## Abstract

**Background/Objectives**: The relationship between ultrasound parameters and fetal health in the context of intrauterine growth restriction (IUGR) pregnancies constitutes a significant focus of scholarly research. A comprehensive range of Doppler and echocardiographic evaluations, encompassing the umbilical artery, middle cerebral artery, ductus venosus, uterine arteries, cardiac contractility, ventricular filling, and the thickness of the interventricular septum, has been proposed in pathological pregnancies. **Methods**: The aim of this paper is to present an examination of these metrics and their implications for fetal health within the framework of IUGR pregnancies and to report a case series in which we analyzed the correlation of these factors. The assessment of these ultrasound indicators can help in better management of the cases in order to obtain better fetal outcomes. **Results**: Our case study presented dynamics corelated to the after-birth evaluation of the neonate, reflecting the importance of complete ultrasound assessment in high-risk cases. **Conclusions**: Speckle tracking echocardiography has significantly advanced our understanding of cardiac function in IUGR fetuses. As shown in our cases, it can be used to detect early signs of cardiac dysfunction, differentiating between FGR and SGA.

## 1. Introduction

Intrauterine growth restriction is a worldwide problem, including developed countries. Its implications exceed the pregnancy, influencing the development of the newborn. The practice of utilizing Doppler ultrasonography in addition to cardiac assessment is essential for the evaluation of fetal health in pregnancies with IUGR. The assessment of middle cerebral artery (MCA) flow dynamics alongside umbilical artery Doppler evaluations is considered an essential marker of fetal health in these pregnancies [1,2].

The study of Roy et al. investigated a series of 50 IUGR pregnancies and found that 70% had abnormal umbilical artery Doppler velocimetry, correlating with higher NICU admissions (71.42%) and neonatal mortality (14.3%) [1]. It is accepted that umbilical artery Doppler is a reliable predictor of fetal compromise and aids in determining the optimal timing for delivery [3,4]. As known, in IUGR, the MCA pulsatility index (PI) is often decreased, indicating increased cerebral blood flow as a compensatory mechanism [5,6]. DV Doppler is a sensitive marker for advanced fetal compromise and is often used in conjunction with other parameters to decide on delivery timing [7].

More recent studies have begun to explore the utility of incorporating advanced imaging techniques, such as three-dimensional (3D) ultrasound and magnetic resonance imaging (MRI), in evaluating IUGR pregnancies. These modalities offer enhanced visualization of fetal anatomy and placental structures. Their role is to reveal underlying pathophysiological changes that traditional two-dimensional ultrasound may not detect. The correct use of 3D ultrasound can provide detailed insights into placental morphology, which is important in order to assess the vascularization and to identify the areas of dysfunction that could lead to compromised fetal growth [1]. Furthermore, MRI has shown promise in evaluating brain development and detecting subtle abnormalities associated with IUGR, thereby guiding more precise management strategies tailored to individual cases [2].

Speckle tracking echocardiography (STE) is an advanced, non-invasive ultrasound technique. It is used to quantify myocardial strain and deformation, to assess cardiac function beyond traditional measures, like left ventricular ejection fraction (LVEF). This method is particularly valuable for assessing both global and regional myocardial performance. STE tracks the movement of speckles in the myocardium to assess local shortening, lengthening, and thickening of heart muscles [8]. STE is used to evaluate left and right ventricular function and can identify early cardiac dysfunction, even when LVEF appears normal, making it useful in various patient populations [8,9]. Global longitudinal strain (GLS) measured by STE is more sensitive than LVEF for detecting left ventricular dysfunction. This finding was transferred to the intrauterine period in order to assess the fetal cardiac function in different pathologies including IUGR.

Fetal STE presents several limitations in assessing fetal cardiac function. Primarily, the limitations are related to technical constraints and fetal conditions. These challenges include issues with frame rate, spatial resolution, and the dependency on angle of insonation, which can affect the accuracy of myocardial deformation measurements. Although STE is considered angle-independent, studies have shown that variations in insonation angles can significantly affect global longitudinal strain (GLS) measurements [10]. Also, low frame rates can lead to inaccurate measurements of myocardial velocity and strain rates [11].

Utilizing advanced ultrasound probes, optimizing imaging settings and establishing standardized imaging protocols and reference values for different gestational ages can improve the results and increase comparability across different studies [12].

The objective of this paper is to present a series of cases with IUGR that were monitored by all mentioned ultrasound parameters and a literature review regarding this topic.

## 2. Materials and Methods

We present two pregnancies diagnosed with IUGR, with gestational age at the start of the screening starting at 27 weeks. At the moment of the first examination, all fetuses were in sinus rhythm and had normal cardiac morphology. Monochorionic twins and all fetuses with malformations, chromosomal abnormalities, cardiac defect or arrhythmia were excluded from the study.

Voluson Expert 22 (BT24) and a Voluson E8 (BT17) ultrasound system equipped with an RM7-C transducer and RAB-6 were used to acquire a high-resolution, zoomed B-Mode of the apical or basal four-chamber view and video acquisition. All equipment used was manufactured by GE HealthCare (Chicago, IL, USA).

The fetal electrophysiological signals were reconstructed with M-mode tracing through the left ventricle lateral wall and septum. In each case the endocardial border of the left ventricle (LV) was identified and manually traced on end diastolic or end systolic frame. The tracing started and ended at the level of insertion of the mitral valve plane. Ulteriorly, the initial trace was manually postprocessed. The TomTEC Arena software (TTA2.51.00) was used for data analysis.

The acquisitions were made in 2D grey-scale cyne-loops images. Images of apical four-chambers were used to obtain the LV longitudinal strain [13]. The quality of the image was optimized depending on the maternal characteristics and the fetal position. The frame rate during the acquisition respected the recommended frame rate in the 30 and 80 frames/s interval [13]. Acquisitions were made on 1–3 cardiac cycles due to fetal movements and variability. All images were performed in the absence of fetal movements, especially fetal respiratory movements.

## 3. Results

We report two cases in which the pregnancy was complicated by IUGR. In both cases, we assess the dynamics of the evolution of ultrasound parameters.

### 3.1. Patient Number 1

Patient number 1 was a 25-year-old patient on the third pregnancy, with two caesarean sections. The patient smoked 2 packs a day during pregnancy. She had no previous history of diabetes or hypertension. The patient was seen from the first trimester of pregnancy. From 26 weeks of pregnancy, the ultrasound showed an SGA fetus with no impact on the vascularization. Serial evaluation is presented in Table 1. At 30 weeks, the fetus was estimated under the 1st percentile with stable parameters and Barcelona stage I. Fetal maturation was realized.

As shown in Table 2, the pulsatility index of the UA, ACM, uterine arteries, and DV were assessed with the help of the Fetal Barcelona Calculator for fetal growth [14]. Speckle tracking echocardiography was used to establish if it is a reliable tool in the assessment of the fetal status. To obtain early diagnosis and estimate the prognostic we performed serial analysis. During evaluation, the values of UA IP increased. The cerebroplacental ratio was kept supraunitary for the first patient (ACM 0.82/UA 0.72). In the meantime, LV endo GLS % maintained values in the normal range, suggesting adequate cardiac function. The LV-GLS was −21.48 at 26 weeks, −28.27 at 28 weeks, and −23.71 at 30 weeks. Considering the normograms (−24.3% ± 3.5%) [15], it was observed that in the evaluation made at 28 weeks, when the cerebroplacental ratio was the lowest (ACM 0.72/UA0.77), the value of LV endo GLS was above the normal range (Table 2). The Barcelona score permitted us to postpone the delivery date.

At 36 weeks, it was decided to deliver the fetus due to acute fetal distress observed on cardiotocography examination. A layer of pericardial fluid was detected during the last ultrasound. The patient gave birth to a female fetus, 1760 g, Apgar index 8, by caesarean section.

In this patient’s case, fetal STE served as a critical tool in evaluating and monitoring fetal cardiac function amidst a diagnosis of small for gestational age and later stage I intrauterine growth restriction. Despite serial ultrasound assessments revealing an estimated fetal growth (EFG) percentile declining to <1% by 30 weeks and an elevated umbilical artery pulsatility index (UA PI), conventional Doppler findings remained stable, with cerebroplacental ratio (CPR) maintained in the supraunitary range throughout most evaluations. This suggested the fetus had not yet developed overt placental insufficiency.

However, fetal cardiac strain analysis provided a more nuanced understanding. LV-GLS remained within the normal physiological range at all evaluations until delivery, consistent with preserved systolic function. These findings sustained the decision to postpone delivery, as they indicated the absence of significant myocardial disfunction despite persistent fetal growth restriction. The associated increase in ejection fraction (EF) and fractional area change (FAC), as well as normal myocardial segmental motion and displacement, further suggested preserved ventricular mechanics at respective stages.

By 36 weeks, however, a shift was observed. The LV-GLS declined markedly to −11.5%, falling below the normative threshold and indicating progressive myocardial strain impairment. This coincided with the development of pericardial effusion and non-reassuring fetal heart rate patterns on cardiotocography (CTG). These cumulative findings prompted the decision to deliver via caesarean section due to acute fetal distress.

In this case, fetal speckle tracking echocardiography provided a sensitive and dynamic assessment of left ventricular performance, offering insights that supplemented traditional Doppler parameters. The ability of LV-GLS to remain stable during early IUGR progression enabled a safe prolongation of pregnancy, while its significant decline at term contributed to the timely identification of cardiac decompensation.

### 3.2. Patient Number 2

Patient number 2 was a 16-year-old primigravida-primipara, that was first evaluated in our clinic at 27 weeks. The patient was a nonsmoker and had no previous history of diabetes or hypertension. During the monitoring period, we observed a decrease in the estimated fetal growth, as seen in Table 3. At 32 weeks, the patient developed IUGR classified as stage I Barcelona.

The ultrasound markers were assessed at each evaluation. The growth curb declined from 15% to 3.1%. According to the Barcelona stage [8], the fetus was considered stage I with the recommendation of follow-up at 1 week. Speckle tracking of the fetal heart revealed the parameters in Table 4. 

**Figure 1:**
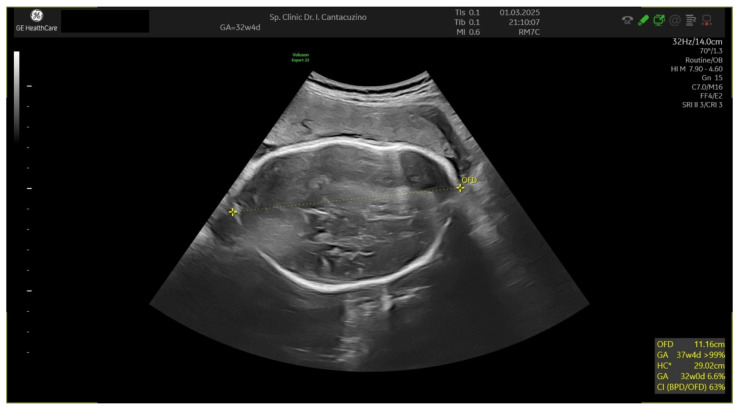
Ultrasound aspect of fetal cranium.

The cerebral-placental ratio was equalized (ACM 0.83/UA 0.83). The LV-GLS was 14.01 at 27 weeks, −19.01 at 29 weeks, and −13.27 at 32 weeks and 2 days (Table 4). In this patient, a mild ventricular asymmetry was observed in favor of the right ventricle and dilated pulmonary artery. No malformation was observed on any of the ultrasounds; thus, it was assumed to be an increased afterload, which was also sustained by the overlaying aspect of the parietal bones (Figure 1) and the impossibility of disengaging the fetus from that fixed position. This would explain the LV endo GLS % outside the normal range.

**Figure 2:**
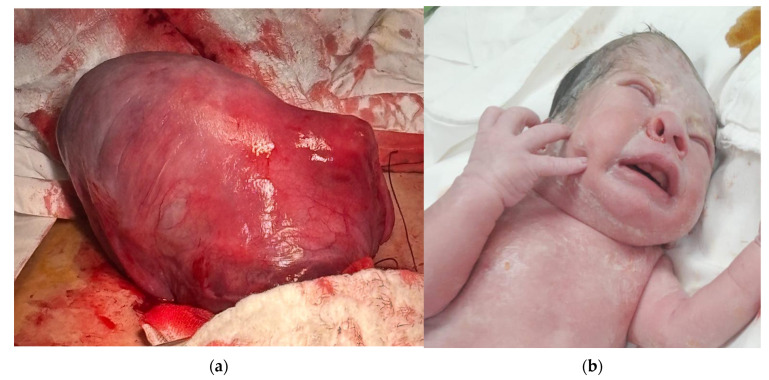
Intraoperative aspect (**a**) Uterine malformation; (**b**) The fetus presented with a deformed cranium and flattened nose due to compression.

**Figure 3:**
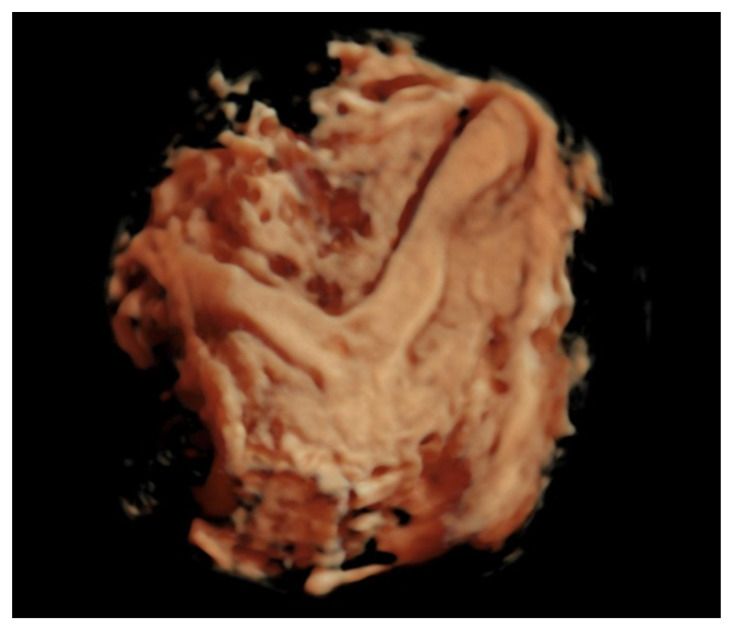
Post-partum ultrasound aspect of the uterus that confirms the suspicion of a bicorn uterus.

Labor began after spontaneous rupture of membrane at 32 weeks and 4 days. Tocolysis was not performed due to premature rupture of the membrane on a fetus in breech position and acute fetal distress. Via caesarean section, the patient delivered a boy, 1930 g and Apgar index 8. During the operation, the presence of a uterine malformation was found, which caused the constriction of the fetal head, thorax, and abdomen (Figure 2). Subsequent ultrasound showed a bicorn uterus (Figure 3). Considering the fetal aspect after birth, the increased afterload suspected during the ultrasound can be explained.

Compensatory mechanisms on the fetal heart are similar to those found in adults; thus, speckle tracking echocardiography can be useful to evaluate the ventricular function in a fetus that experiences increased afterload. The possibility of exact quantification, standardization and impact on the management of these measurements needs further research. For this particular case, the IUGR in combination with significant LV-GLS modification suggested an external mechanical factor represented by the constrictive uterus.

From the initial evaluation at 27 weeks, LV-GLS was severely impaired, suggesting an abnormal contractile pattern. These abnormal strain values pointed to increased afterload and potential preload reduction, well before conventional Doppler parameters would typically suggest cardiac decompensation. Supporting this interpretation, ventricular asymmetry in favor of the right ventricle was detected alongside a dilated pulmonary artery (Z score = 3.24, Main pulmonary artery diameter = 7.82 mm)—findings that further hinted at altered loading conditions rather than primary myocardial pathology. In addition, segmental strain values and displacement parameters demonstrated inconsistent and aberrant myocardial mechanics, especially at the apical and mid-septal levels. Reductions in MAPSE and fractional area change (FAC), as well as abnormal left ventricular volume dynamics (decreased EDV and inconsistent ESV), strengthened the suspicion of a non-standard stressor impacting cardiac function.

Despite IUGR being diagnosed as Barcelona stage I, the significant and disproportionate LV-GLS abnormalities, together with stable or mildly altered Doppler findings (e.g., cerebroplacental ratio of 1.0), suggested an underlying mechanical constraint rather than isolated placental dysfunction. This hypothesis was confirmed at delivery when a bicornuate uterus was discovered, with evidence of fetal compression affecting the head, thorax, and abdomen—correlating with the suspected increase in afterload seen on fetal echocardiography.

## 4. Discussion

Fetal cardiac function can be impaired in IUGR due to increased afterload and hypoxemia. The effects include ventricular contractility and diastolic filling. Studies using speckle tracking echocardiography (STE) have shown reduced global longitudinal strain and altered ventricular filling patterns in IUGR fetuses [16,17,18,19]. The E/A ratio (early wave to atrial wave ratio) of trans-mitral flow is lower in IUGR fetuses, indicating diastolic dysfunction [20]. Assessment of ventricular function can evaluate subclinical cardiac dysfunction and help predict adverse outcomes [17,18,19]. The IVS thickness is measured to assess fetal cardiac adaptation to increased afterload in IUGR. Increased IVS thickness has been observed in IUGR fetuses, likely as a compensatory mechanism to increased placental resistance [6]. A study of 75 high-risk pregnancies found that IVS thickness was significantly larger in IUGR cases compared to controls [6].

### 4.1. The Fetal Heart in IUGR

Fetuses with IUGR often exhibit abnormal cardiac contractility, particularly in the left ventricle (LV), which may demonstrate decreased global contractility compared to controls. The left ventricle can show modified transverse and longitudinal contractility. The compromised systolic function can be a sign of altered myocardial performance. As is known, the abnormal umbilical artery pulsatility index can correlate with changes in the fetal heart’s structure and function. This type of change in the left ventricle can lead to a higher incidence of ventricular area abnormalities. Studies have shown that the size of the fetal heart is a more reliable indicator of fetal health compared to gestational age, in cases of abnormal growth.

Previous research has indicated that the RV/LV area ratio can be disproportionately affected early in the growth-restricted process. Fetuses with IUGR show a significantly increased rate of atypical left ventricular (LV) contractility compared to standard fetuses, emphasizing a crucial issue for fetal health [16,21]. Left ventricular contractility abnormalities are observed in a notable percentage of IUGR cases, although these are less pronounced than those seen in the LV, suggesting a differential impact on cardiac function [16,21].

Devore [22,23] evaluated the fetal heart in the context of intrauterine growth restriction. His study [22] evaluated left ventricular (LV) size and function in 200 healthy fetuses, showing a strong correlation between ultrasound-derived gestational age and last menstrual period gestational age. The mean fetal heart rate was 144 beats per minute, with a mean image acquisition frame rate of 109 Hz [23]. The analysis revealed R^2^ values for end-diastolic volume (EDV), end-systolic volume (ESV), stroke volume (SV), and cardiac output (CO) ranging from 0.71 to 0.78. Five fetuses at risk for abnormal LV function exhibited expected pathologic conditions [23]. It seems that the prevalence of abnormal fetal heart size, rather than gestational age, is a more reliable indicator of fetal health in IUGR, emphasizing the need for accurate assessments of estimated fetal weight [16].

DeVore [15] measured end-diastolic ventricular area, basal-apical length, and transverse widths in normal fetuses vs. fetuses with cardiac anomalies and found a significant correlations with somatic and age-independent variables, particularly head circumference and estimated fetal weight, with R^2^ values between 0.63 and 0.85.

Research has indicated that IUGR not only correlates with immediate fetal heart dysfunction but also causes a predisposition to chronic cardiovascular diseases during adulthood. Furthermore, the identification of specific echocardiographic parameters, such as isovolumetric relaxation time, could serve as vital indicators for clinicians to monitor and address potential risks associated with impaired fetal development [17].

A multiparametric approach, combining Doppler, echocardiographic, and biophysical markers, provides a more comprehensive assessment of fetal well-being.

The biophysical profile (BPP) is the main assessment tool in obstetrics, designed to evaluate fetal well-being through five key parameters: fetal heart rate reactivity, fetal breathing movements, gross body movements, fetal tone, and amniotic fluid volume [18]. Conversely, although some research suggests that while the BPP is widely used, its efficacy in improving outcomes in high-risk pregnancies remains debated [19], it remains an important tool in the assessment of the fetal wellbeing [20].

Studies have shown that combining umbilical artery, MCA, and DV Doppler with cardiac function parameters (global strain, IVS thickness) can have the benefit of improving the prediction of unfavorable outcomes [11,13,14,21].

### 4.2. 2D vs. 3D in Fetal Heart Speckle Tracking

Speckle tracking echocardiography has emerged as a valuable tool for assessing fetal cardiac function, offering both 2D and 3D imaging modalities. There are differences in the acquisition techniques and the use of resulting data. Two-dimensional speckle tracking is less dependent on the angle of insonation, making it more versatile for fetal heart assessments [24,25,26,27]. Studies have shown high intraobserver and interobserver reproducibility for parameters like ventricular area and global strain, particularly in the left ventricle [17,28,29]. Two-dimensional speckle tracking has been successfully used to evaluate fetal cardiac function in both normal and pathological conditions, including fetal growth restriction and congenital heart disease [29,30,31].

Despite its advantages, 2D speckle tracking has certain limitations. Higher frame rates improve the accuracy of strain measurements, though some parameters, like mechanical synchrony, are less affected by frame rate variations [32]. The quality of image acquisition and operator expertise significantly impact the reliability of 2D speckle tracking results [27].

The use of 3D speckle tracking offers several advantages. Three-dimensional speckle tracking provides a more complete evaluation of both global and segmental myocardial function, including parameters, like area strain [31,33]. This technique can provide the assessment of the regional wall motion abnormalities, offering additional insights into left ventricular mechanics [31,34]. Unfortunately, 3D imaging requires higher-quality images and is more sensitive to artifacts, which can complicate data acquisition and analysis [33,35]. The normal limits and cut-off values for 3D strain parameters are highly vendor-specific, limiting standardization across different ultrasound systems [33].

### 4.3. Speckle Tracking in IUGR

STE has been shown to detect subclinical cardiac dysfunction in IUGR fetuses. Studies have demonstrated that IUGR fetuses exhibit reduced global longitudinal strain (GLS) and other strain parameters compared to appropriate-for-gestational-age (AGA) fetuses. For instance, one study found that the absolute values of myocardial global longitudinal strain (myoGLS) and endocardial global longitudinal strain (endoGLS) were significantly lower in IUGR fetuses than in AGA fetuses [36,37]. This suggests that STE can identify early signs of cardiac remodeling in IUGR fetuses, even before clinical symptoms become apparent.

Fetal growth restriction (FGR) and small-for-gestational-age (SGA) are often used interchangeably, but they represent distinct conditions. FGR is characterized by a pathologic restriction of growth, while SGA refers to fetuses that are constitutionally small but otherwise healthy. STE has been used to differentiate between these two groups. For example, one study found that FGR fetuses had significantly lower GLS values compared to SGA fetuses, indicating more severe cardiac dysfunction in FGR [36,38]. This differentiation is critical for clinical management and prognosis.

The prognostic value of STE in IUGR has been well-documented. Studies have shown that abnormal strain parameters, such as reduced GLS, are associated with adverse perinatal outcomes, including preterm delivery, low birth weight, and neonatal morbidity. For instance, one study found that a cut-off value of 0.54 for the modified myocardial performance index (MPI) conferred a sensitivity of 87% and specificity of 75% for predicting adverse outcomes in IUGR fetuses [39,40]. These findings highlight the potential of STE to guide clinical decision-making and improve outcomes in IUGR pregnancies.

**Figure 4:**
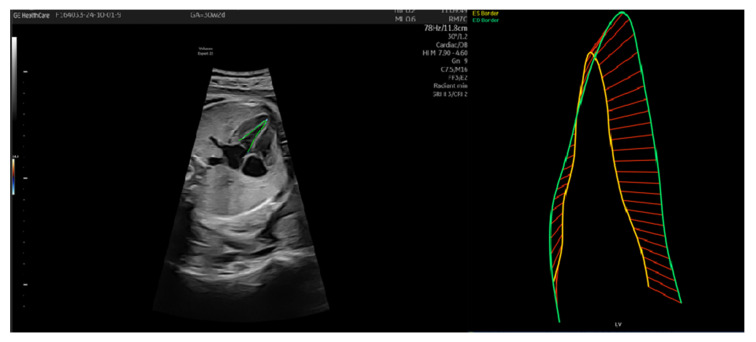
Patient 1—Left ventricle speckle tracking. The yellow line represents the endocardial border in systole; green line represents endocardial border in diastole and the red lines are graphic representations of the distance traveled in time by the border. FAC was calculated by subtracting LVSA from LVDA and dividing it by LVDA times 100 ($\frac{LVDA-LVSA}{LVDA}\times 100\;$).

In the first case, we had a patient with IUGR, Barcelona stage I from 28 weeks with normal strain until delivery at 36 weeks. Although it was a severe IUGR, the cardiac function was preserved (Figure 4).

**Figure 5:**
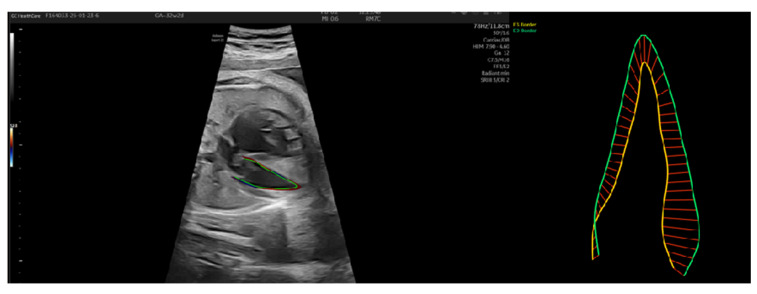
Patient 2—Left ventricle speckle tracking. The yellow line represents the endocardial border in systole; green line represents endocardial border in diastole and the red lines are graphic representations of the distance traveled in time by the border. FAC was calculated by subtracting LVSA from LVDA and dividing it by LVDA times 100 ($\frac{LVDA-LVSA}{LVDA}\times 100\;$).

In the second case, the values of GLS endo were decreased, suggesting cardiac remodeling and perturbed left ventricle function. These changes were due to increased afterload due to compression of fetal body parts (Figure 5).

### 4.4. Current Status and Perspectives in IUGR

The integration of Doppler ultrasonography with biophysical assessments, such as biophysical profile and cardiotocography, is emphasized in current guidelines for monitoring fetal growth. This combined approach leads to identifying fetuses at risk of adverse outcomes, particularly in cases of intrauterine growth restriction (IUGR) [41].

The ISUOG and FIGO guidelines advocate for the use of Doppler alongside BPP or cCTGF to improve monitoring accuracy and management strategies. Future studies should focus on standardizing the protocols for combined assessments to ensure consistency in clinical practice [42,43]. Incorporating fetal speckle tracking echocardiography (STE) into current protocols for assessing intrauterine growth restriction (IUGR) alongside biophysical scores and cardiotocography (CTG) presents several potential benefits. The main aspects are represented by the fact that STE can identify subclinical cardiac dysfunction in fetuses with IUGR and GLS is more sensitive than conventional ejection fraction metrics. It is possible that in selected cases, by integrating STE with biophysical profiles and CTG, clinicians can achieve a comprehensive assessment of fetal status, thus enhancing decision-making processes.

## 5. Conclusions

Speckle tracking echocardiography has significantly advanced our understanding of cardiac function in IUGR fetuses. It is important in selected cases to perform a complete examination of the fetus including largely used parameters, like Doppler on the uterine artery, umbilical artery, and medium cerebral artery, but also advanced parameters, like intraventricular septum size and ventricular function. These parameters can help to detect early signs of cardiac dysfunction, differentiate between FGR and SGA, and predict adverse outcomes. As the review states, the use of speckle tracking of the fetal heart can be a valuable tool in the clinical management of IUGR pregnancies. As research continues to refine the use of STE in fetal cardiology, its role in improving perinatal outcomes is likely to expand further.

## Figures and Tables

**Table 1:** Ultrasound assessment of the fetal grow for patient no. 1.

Gestational Age (Weeks)	26 Weeks	28 Weeks	30 Weeks
EGF	737	994	1093
Hadlock %	4.1	2.2	<1
BPD	6.18	6.66	6.9
HC	23.31	25.11	25.96
AC	19.85	23.83	25.94
FL	4.52	4.98	5.29
AFI modified	no	no	no
Barcelona index	no	Barcelona stage I	Barcelona stage I
IVS thickness (mm)	1.41	2.56	2.45

**Table 2:** Ultrasound markers for patient no. 1.

Patient Number 1	26 Weeks	28 Weeks	30 Weeks	36 Weeks
UA IP	1.02	1.25	1.26	1.30
UA IR	0.66	0.72	0.72	0.74
reverse flow UA	no	no	no	No
MCA IP	1.72	1.65	1.91	1.64
MCA IR	0.81	0.77	0.82	0.75
DV IP	0.61	no	no	No
right Ut A IP	0.77	0.96	0.83	0.79
left Ut A IP	1.12	0.87	1.19	1.1
LV endo GLS %	−21.48	−28.27	−23.71	−11.5
EF %	62.53	70.93	72.76	69.1
LV EDA cm^2^	0.9	1.25	1.32	1.39
LV ESA cm^2^	0.49	0.53	0.58	0.71
LV ESL cm	1.35	1.57	1.64	1.9
ESD bas cm	0.53	0.7	0.69	0.7
LV ESD mid cm	0.39	0.42	0.35	0.3
LV EDL cm	1.72	2.16	2.13	2.1
LV EDD bas cm	0.73	0.71	0.89	1.0
LV EDD mid cm	0.58	0.67	0.73	0.7
LV EDV ml	0.44	0.68	0.74	0.8
LV ESV ml	0.16	0.2	0.2	0.27
MAPSE l (lateral mitral annulus) cm	0.37	0.53	0.39	0
MAPSE s (septal mitral annulus) cm	0.39	0.64	0.6	0.4
FAC (fractional area change) %	45.95	57.31	56.09	49.3
basal inf/sept peak value % (longitudinal strain)	232	218	77	208
mid inf/sept peak value	232	205	90	390
apical septal peak value	155	256	192	364
apical lateral peak value	144	307	154	364
mid ant/lat peak value	221	205	179	364
basal ant/lats peak value	232	218	102	364
basal inf/sept longitudinal displacement (mm)	232	205	269	-
mid inf/sept longitudinal displacement	122	141	269	-
apical septal longitudinal displacement	88	26	1	-
apical lateral longitudinal displacement	11	461	90	-
mid ant/lat longitudinal displacement	111	320	282	-
basal ant/lat longitudinal displacement	232	192	269	-

**Table 3:** Ultrasound assessment of the fetal grow for patient no. 2.

Gestational Age (Weeks)	27 Weeks	29 Weeks	32 Weeks 2 Days	32 Weeks 4 Days
EGF	967	1186	1548	1560
Hadlock %	15	12	4.1	3.1
BPD	5.96	6.43	6.76	7.08
HC	25.49	26.8	28.7	29.35
AC	22.2	23.83	25.94	25.95
FL	4.99	5.37	5.97	5.87
AFI modified	no	no	no	no
Barcelona index	no	no	Barcelona stage I	Barcelona stage I
IVS thickness (mm)	1.91	2.14	4.03	4

**Table 4:** Ultrasound markers for patient no. 2.

Patient Number 2	27 Weeks	29 Weeks	32 Weeks 2 Days	32 Weeks 4 Days
UA IP	1.98	1.81	1.77	1.2
UA IR	0.72	0.86	0.82	0.83
reverse flow UA	no	no	no	no
MCA IP	1.94	2.22	1.64	1.98
MCA IR	0.86	0.88	0.8	0.83
DV IP	no	no	no	0.83
right Ut A IP	no	no	no	0.95
left Ut A IP	no	no	no	1.72
LV endo GLS %	14.01	−19.01	−13.27	−10.14
EF %	−32.93	64.4	63.8	49.57
LV EDA cm^2^	0.91	1.4	1.95	1.32
LV ESA cm^2^	1.13	0.76	1.08	0.83
LV ESL cm	1.59	1.31	2.27	1.85
ESD bas cm	0.97	0.84	0.88	0.87
LV ESD mid cm	0.73	0.61	0.53	0.48
LV EDL cm	1.42	1.54	2.59	2.03
LV EDD bas cm	0.93	1.1	0.95	0.81
LV EDD mid cm	0.73	1	0.9	0.78
LV EDV mL	0.51	1.08	1.37	0.75
LV ESV mL	0.68	0.38	0.5	0.38
MAPSE l (lateral mitral annulus) cm	0.13	0.25	0.41	0.21
MAPSE s (septal mitral annulus) cm	0.22	0.27	0.23	0.14
FAC (fractional area change) %	−24.7	45.72	44.36	36.99
basal inf/sept peak value % (longitudinal strain)	381	160	218	231
mid inf/sept peak value	417	135	141	179
apical septal peak value	417	160	218	154
apical lateral peak value	417	147	218	141
mid ant/lat peak value	417	147	128	179
basal ant/lats peak value	417	197	192	269
basal inf/sept longitudinal displacement (mm)	12	197	103	179
mid inf/sept longitudinal displacement	37	209	269	320
apical septal longitudinal displacement	37	221	26	333
apical lateral longitudinal displacement	307	197	77	77
mid ant/lat longitudinal displacement	320	147	116	141
basal ant/lat longitudinal displacement	357	160	128	154

## Data Availability

The data presented in this study are available on request from the corresponding author due to ethical reasons.

## Abbreviations

The following abbreviations are used in this manuscript:

IUGR	Intrauterine growth restriction
LV	Left ventricle
RV	Right ventricle
BPD	Biparietal diameter
HC	Head circumference
AC	Abdominal circumference
FL	Femur length
AFI	Amniotic fluid index
IVS	Interventricular sept
UA	Umbilical artery
IR	Resistance index
IP	Pulsatility index
MCA	Medial cerebral artery
DV	Ductus venosus
Ut A	Uterine artery
GLS	Global longitudinal strain
EF	Ejection fraction
LV EDA	LV end-diastolic area: Cross-sectional area of the LV at end-diastole.
LV ESA	LV end-systolic area: Cross-sectional area of the LV at end-systole.
LV ESL	LV end-systolic length: Length of LV cavity during systole.
ESD bas	End-systolic diameter (basal)
LV ESD	Left Ventricular End-Systolic Diameter
LV EDL	Left Ventricular End-Diastolic Length
LV EDD	Left Ventricular End-Diastolic Diameter
LV EDV	Left Ventricular End-Diastolic Volume
LV ESV	Left Ventricular End-Systolic Volume
MAPSE	Mitral annulus Plane Systolic Excursion
FAC	Fractional area change

## References

[1] Rao S.V., Venkateswarlu B. (2016). Role of doppler sonography in clinically suspected iugr pregnencies: A descriptive study. Med. Sci..

[2] Rautray P., Swain B. (2016). Ultrasonography with doppler assessment of the fetus with Intrauterine Growth Restriction (IUGR). Indian J. Community Fam. Med..

[3] Das P.K., Das S., Chakrabarti S. (2017). Role of doppler ultrasound in prediction of perinatal outcome in IUGR. J. Évid. Based Med. Healthc..

[4] Botsis D., Vrachnis N., Christodoulakos G. (2006). Doppler Assessment of the Intrauterine Growth-Restricted Fetus. Ann. N. Y. Acad. Sci..

[5] Pasupuleti B., Narra R., Jukuri N., Kamaraju S.K., Sushmitha S. (2015). Colour doppler evaluation of pregnancy induced hyper-tension and iugr. Int. J. Biol. Med. Res..

[6] Hssan H.G.E.M.A., El Wahed M.A.A., Aziz M.M.A. (2022). Interventricular Septal Thickness and Doppler Indices as Multiparametric Assessment of High-Risk Pregnancy and Their Relation to Fetal Outcome. Egypt. J. Hosp. Med..

[7] Machlitt A., Wauer R.R., Chaoui R. (2001). Longitudinal observation of deterioration of Doppler parameters, computerized cardiotocogram and clinical course in a fetus with growth restriction. J. Perinat. Med..

[8] Morissens M., Rodriguez J.C., Azerad M., Besse-Hammer T., Efira A. (2018). Added value of speckle tracking in the evaluation of cardiac function in patients with sickle cell disease. Br. J. Haematol..

[9] Amer S.M., El Nemr S.B., El Amrosy D.M., Elrasol O.A. (2024). Speckle tracking echocardiography of right and left ventricles in children of atrial septal defect and ventricular septal defect. Tanta Med. J..

[10] Nichting T.J., de Vet C.M., van der Ven M., van der Woude D.A., van Sloun R.J., Oei S.G., van Oostrum N.H., van Laar J.O.H. (2022). Angle Independency of Fetal Speckle-Tracking Echocardiography: A Commentary Letter. J. Am. Soc. Echocardiogr..

[11] Day T.G., Charakida M., Simpson J.M. (2019). Using speckle-tracking echocardiography to assess fetal myocardial deformation: Are we there yet?. Ultrasound Obstet. Gynecol..

[12] Young K., Hooton C., Zimmerman M.B., Reinking B., Gupta U. (2023). Fetal left and right ventricular strain parameters using speckle tracking in congenital heart diseases. Int. J. Cardiovasc. Imaging.

[13] Bansal M., Kasliwal R.R. (2013). How do I do it? Speckle-tracking echocardiography. Indian Heart J..

[14] García-Otero L., Gómez O., Rodriguez-López M., Torres X., Soveral I., Sepúlveda-Martínez Á., Guirado L., Valenzuela-Alcaraz B., López M., Martínez J.M. (2020). Nomograms of Fetal Cardiac Dimensions at 18–41 Weeks of Gestation. Fetal Diagn. Ther..

[15] Akazawa Y., Yasukochi S., Takei K., Takigiku K., Inamura N., Takagi K., Pooh R.K., Yoshimatsu J., Kamei Y., Tamaru S. (2024). Normal values and distribution of ventricular global longitudinal strain in 513 healthy fetuses measured by two-dimensional speckle-tracking echocardiography: A multi-institutional cohort study. Heart Vessel..

[16] Girsen A., Ala-Kopsala M., Mäkikallio K., Vuolteenaho O., Räsänen J. (2007). Cardiovascular hemodynamics and umbilical artery N-terminal peptide of proB-type natriuretic peptide in human fetuses with growth restriction. Ultrasound Obstet. Gynecol..

[17] Änghagen O., Engvall J., Gottvall T., Nelson N., Nylander E., Bang P. (2022). Developmental Differences in Left Ventricular Strain in IUGR vs. Control Children the First Three Months of Life. Pediatr. Cardiol..

[18] Sapoval J., Singh V., Carter R.E. (2025). Ultrasound Biophysical Profile (Archived). StatPearls [Internet].

[19] Lalor J.G., Fawole B., Alfirevic Z., Devane D. (2008). Biophysical profile for fetal assessment in high risk pregnancies. Cochrane Database Syst. Rev..

[20] Baschat A.A., Galan H.L., Lee W., DeVore G.R., Mari G., Hobbins J., Vintzileos A., Platt L.D., Manning F.A. (2022). The role of the fetal biophysical profile in the management of fetal growth restriction. Am. J. Obstet. Gynecol..

[21] Huntley E.S., Hernandez-Andrade E., Soto E., DeVore G., Sibai B.M. (2021). Novel Speckle Tracking Analysis Showed Excellent Reproducibility for Size and Shape of the Fetal Heart and Good Reproducibility for Strain and Fractional Shortening. Fetal Diagn. Ther..

[22] Mazzeo S., Scalia M., Zamagni G., Bussolaro S., Ricci G., Greco P., Stampalija T. (2023). EP01.01: Speckle-tracking echocardiography: Fetal cardiac parameters in fetuses with ventricular chamber disproportion and neonatal outcome. Ultrasound Obstet. Gynecol..

[23] Gores G., Raith W., Ravekes W., Koestenberger M. (2014). Relevance of longitudinal systolic heart function in fetuses with intrauterine growth restriction. Ultrasound Obstet. Gynecol..

[24] Tsyv’Yan P.B., Bashmakova N.V., Artem’Eva O.G., Panacheva N.M., Orekhova V.K., Protsenko Y.L. (2004). Contractile Cardiac Activity in Fetuses with Intrauterine Growth Retardation: Correlations between Regional Nonuniformity, Relaxation, and Afterload. Hum. Physiol..

[25] Nichting T.J., de Vet C.M., van der Ven M., van der Woude D.A.A., Regis M., van Sloun R.J.G., Oei S.G., van Laar J.O.E.H., van Oostrum N.H.M. (2023). The impact of angles of insonation on left and right ventricular global longitudinal strain estimation in fetal speckle tracking echocardiography. PLoS ONE.

[26] DeVore G.R., Klas B., Satou G., Sklansky M. (2018). Evaluation of Fetal Left Ventricular Size and Function Using Speckle-Tracking and the Simpson Rule. J. Ultrasound Med..

[27] DeVore G.R., Cuneo B., Klas B., Satou G., Sklansky M. (2018). Comprehensive Evaluation of Fetal Cardiac Ventricular Widths and Ratios Using a 24-Segment Speckle Tracking Technique. J. Ultrasound Med..

[28] Willruth A., Geipel A., Merz W., Gembruch U. (2012). Speckle tracking—Ein neues Ultraschallverfahren zur Beurteilung der fetalen Myokardfunktion. Z. Geburtshilfe Neonatol..

[29] Ta-Shma A., Perles Z., Gavri S., Golender J., Tarshansky S., Shlichter C., Bar Tov H., Rein A.J. (2008). Analysis of Segmental and Global Function of the Fetal Heart Using Novel Automatic Functional Imaging. J. Am. Soc. Echocardiogr..

[30] Im D., Kim Y., Park J., Ahn K., Won H., Kwon J., Nam G., Seong W., Lee K., Hong S. (2023). EP01.31: Evaluation of fetal heart function using fetal speckle-tracking echocardiography: Korean multicentre prospective study. Ultrasound Obstet. Gynecol..

[31] Muraru D., Niero A., Rodriguez-Zanella H., Cherata D., Badano L. (2018). Three-dimensional speckle-tracking echocardiography: Benefits and limitations of integrating myocardial mechanics with three-dimensional imaging. Cardiovasc. Diagn. Ther..

[32] Barker P.C., Houle H., Li J.S., Miller S., Herlong J.R., Camitta M.G. (2009). Global Longitudinal Cardiac Strain and Strain Rate for Assessment of Fetal Cardiac Function: Novel Experience with Velocity Vector Imaging. Echocardiography.

[33] Haeger C., Hammer K., Braun J., Oelmeier K., Köster H.A., Möllers M., Koch R., Steinhard J., Klockenbusch W., Schmitz R. (2021). Importance of frame rate for the measurement of strain and synchrony in fetuses using speckle tracking echocardiography. J. Perinat. Med..

[34] Maffessanti F., Nesser H.-J., Weinert L., Steringer-Mascherbauer R., Niel J., Gorissen W., Sugeng L., Lang R.M., Mor-Avi V. (2009). Quantitative Evaluation of Regional Left Ventricular Function Using Three-Dimensional Speckle Tracking Echocardiography in Patients With and Without Heart Disease. Am. J. Cardiol..

[35] de Isla L.P., Vivas D., Zamorano J. (2008). Three-dimensional speckle tracking. Curr. Cardiovasc. Imaging Rep..

[36] Piveta R.B., Aguiar M.O.D., da Silva L.L.M., Vieira M.L.C. (2024). My Approach to 3-Dimensional Left Ventricular Strain. ABC Imagem Cardiovasc..

[37] Zhang W., Zhang B., Wu T., Li Y., Qi X., Tian Y., Chen J., Luo H. (2023). Value of two-dimensional speckle-tracking echocardiography in evaluation of cardiac function in small fetuses. Quant. Imaging Med. Surg..

[38] Sharma B., Verma A., Meena C., Gurjar A., Chakraborty A., Srivastav A. (2019). Assessment of the Cardiac Function in Intrauterine Growth-Restricted Fetuses and Appropriate for Gestational Age Fetuses. J. Obstet. Gynecol. India.

[39] Ren Y., Zhu C. (2023). OC17.01: Clinical value of 2D speckle tracking for the evaluation of ventricular global longitudinal strain in low-risk fetuses and SGA fetuses. Ultrasound Obstet. Gynecol..

[40] Bhorat I.E., Bagratee J.S., Pillay M., Reddy T. (2014). Determination of the myocardial performance index in deteriorating grades of intrauterine growth restriction and its link to adverse outcomes. Prenat. Diagn..

[41] Salomon L., Alfirevic Z., Da Silva Costa F., Deter R., Figueras F., Ghi T., Glanc P., Khalil A., Lee W., Napolitano R. (2019). ISUOG Practice Guidelines: Ultrasound assessment of fetal biometry and growth. Ultrasound Obstet. Gynecol. Off. J. Int. Soc. Ultrasound Obstet. Gynecol..

[42] Melamed N., Baschat A., Yinon Y., Athanasiadis A., Mecacci F., Figueras F., Berghella V., Nazareth A., Tahlak M., McIntyre H.D. (2021). FIGO (International Federation of Gynecology and Obstetrics) initiative on fetal growth: Best practice advice for screening, diagnosis, and management of fetal growth restriction. Int. J. Gynecol. Obstet..

[43] Lees C., Stampalija T., Baschat A.A., da Silva Costa F., Ferrazzi E., Figueras F., Hecher K., Kingdom J., Poon L.C., Salomon L.J. (2020). ISUOG Practice Guidelines: Diagnosis and management of small-for-gestational-age fetus and fetal growth restriction. Ultrasound Obstet. Gynecol..

